# Metastatic Burden Defines Clinically and Biologically Distinct Subgroups of Stage 4 High-Risk Neuroblastoma

**DOI:** 10.3390/jcm9092730

**Published:** 2020-08-24

**Authors:** Eun Seop Seo, Eun-jin Lee, Boram Lee, Muheon Shin, Young-Seok Cho, Ju Kyung Hyun, Hee Won Cho, Hee Young Ju, Keon Hee Yoo, Hong Hoe Koo, Ji Won Lee, Ki Woong Sung

**Affiliations:** 1 Department of Pediatrics, Samsung Medical Center, Sungkyunkwan University School of Medicine, Seoul 06351, Korea; eunseop720@gmail.com (E.S.S.); jukyung.hyun@samsung.com (J.K.H.); hw87.cho@samsung.com (H.W.C.); heeyoung.ju@samsung.com (H.Y.J.); keonhee.yoo@samsung.com (K.H.Y.); hhkoo.koo@samsung.com (H.H.K.); 2 Samsung Genome Institute, Samsung Medical Center, Seoul 06351, Korea; eunjin_lee@samsung.com (E.-j.L.); Irazim@gmail.com (B.L.); 3 Department of Nuclear Medicine, Samsung Medical Center, Sungkyunkwan University School of Medicine, Seoul 06351, Korea; muheon.shin@samsung.com (M.S.); ysnm.cho@samsung.com (Y.-S.C.)

**Keywords:** high-risk neuroblastoma, metastasis, prognosis, tandem transplant

## Abstract

This study aimed to identify the prognostic subgroups of stage 4 high-risk neuroblastoma based on metastatic burden and explore their distinct clinical and genomic features. Patients aged ≥18 months with stage 4 and metaiodobenzylguanidine-avid neuroblastoma were enrolled. One hundred and thirty eligible patients were treated under the tandem high-dose chemotherapy scheme. Prognostic significance of metastatic burden measured by the modified Curie score was analyzed using a competing risk approach, and the optimal cut-point was determined. Metastasis-specific subgroups (cut-point: 26) were compared using clinicopathological variables, and differential gene expression analysis and gene set variation analysis (GSVA) were performed using RNA sequencing (RNA-seq). Metastatic burden at diagnosis showed a progressive association with relapse/progression. After applying the cut-point, patients with high metastatic burden showed >3-fold higher risk of relapse/progression than those with low metastatic burden. Moreover, patients with high metastatic burden showed smaller primary tumors and higher biochemical marker levels than those with low metastatic burden. In the genomic analysis, 51 genes were found to be differentially expressed based on the set criteria. GSVA revealed 55 gene sets, which significantly distinguished patients with high metastatic burden from those with low metastatic burden at a false discovery rate <0.25. The results indicated the prognostic significance of metastatic burden in stage 4 high-risk neuroblastoma, and we identified the distinct clinicopathological and genomic features based on metastatic burden. This study may aid in the better understanding and risk-stratification of stage 4 high-risk neuroblastoma patients.

## 1. Introduction

Few tumors have been recognized to show as many diverse clinical presentations and biological features as neuroblastoma (NB) [1]. NB is characterized by heterogenous clinical behaviors, ranging from spontaneous regression to inexorable progression [2]. Thus, considerable efforts have been made to develop risk-stratification based on the known prognostic factors, and the results of these efforts have guided physicians in therapeutic decision-making and prognostication [3]. However, >50% of the children classified as high-risk show disease progression or relapse, despite intensive multimodal therapy. Therefore, there is an urgent need to define the subgroups of high-risk NB with inferior outcomes [4].

Previous studies have suggested that the extent of metastasis affects the prognosis of high-risk NB patients [5,6,7,8]. An increasing number of metastatic compartments are related to poor outcome [6], and the extent of metaiodobenzylguanidine (mIBG)-avid disease is prognostic [5,8,9]. However, whether metastatic burden at diagnosis has prognostic significance is controversial, and whether its prognostic impact persists in the context of tandem high-dose chemotherapy and autologous stem cell transplantation (HDCT/auto-SCT) remains unclear. Moreover, the clinical behavior and underlying biology driving these prognostic differences must be elucidated.

In this comprehensive evaluation of high-risk NB, we investigated the prognostic significance of metastatic burden at diagnosis of high-risk stage 4 NB patients uniformly treated according to the protocols where tandem HDCT/auto-SCT was the essence [10,11,12]. Furthermore, we analyzed the differences in clinical characteristics and gene expression profiling, arising from metastatic burden of varying degrees, thus providing insights into NB.

## 2. Methods

### 2.1. Patient Cohort and Data Collection

High-risk NB patients were enrolled if they met the following inclusion criteria: stage 4 NB diagnosed based on the International Neuroblastoma Staging System criteria [13]; age at diagnosis ≥18 months; diagnosed between January 2004 and August 2019; and mIBG-avid disease. We excluded patients who underwent delayed (>30 days after diagnosis) or no initial mIBG scan to avoid effects of chemotherapy. The Institutional Review Board at the Samsung Medical Center (SMC) approved this study (NO. SMC 2015-11-053 and SMC 2020-04-200). Tissue samples for genomic analyses, along with written informed consent obtained from the parents or legal guardians of the patient, were collected during the diagnostic procedure. The data for clinical variables, including age, primary site, tumor volume, histological classification, *MYCN* amplification (MNA), chromosomal aberration, and biochemical markers, such as 24 h urine vanillylmandelic acid (VMA), were collected through review of medical records. The equation, volume = (π/6) × depth × width × height, was used for tumor volume measurement [14].

### 2.2. Treatment

The treatment, according to the SMC NB-2004, -2009, and -2014 protocols, included nine cycles of induction chemotherapy, followed by tandem HDCT/auto-SCT, local radiation therapy, and subsequent 13-*cis*-retinoic acid treatment with or without subcutaneous interleukin-2 treatment, as described in Supplemental Table S1 [10,11]. Surgical resection of the primary tumor was recommended during induction chemotherapy, and was usually performed after six cycles of induction chemotherapy. Patients without progression subsequently underwent tandem HDCT/auto-SCT with carboplatin, etoposide, and cyclophosphamide (CEC), followed by thiotepa and melphalan, after completion of induction therapy. While total-body irradiation (TBI) was incorporated into the second HDCT/auto-SCT regimen before 2009, high-dose ^131^I-mIBG treatment replaced TBI in the second HDCT/auto-SCT regimen from 2009 onwards. Based on the tumor response, CEC dose adjustment and ^131^I-mIBG treatment were attempted according to the SMC NB-2014 protocol. None of the patients received anti-ganglioside (GD)-2 antibody which is an advanced immunotherapeutic modality as first-line therapy.

### 2.3. Assessment of Metastasis

^123^I-mIBG (from 2009) or ^131^I-mIBG (before 2009) scintigraphy was performed to assess the extent of metastasis at initial evaluation, following the recommended guidelines [15]. After intravenous injection of ^131^I-mIBG or ^123^I-mIBG (dose was adjusted by weight), planer images were obtained. Each scan was assessed for mIBG avidity at nine skeletal sites (head, chest, T-spine, L-spine, pelvis, upper arms, lower arms, femurs, and lower legs) and additional extraosseous lesions. All the scans were scored by two nuclear physicians using the modified Curie score. Computed tomography (CT) and/or magnetic resonance imaging, ^18^F-fluoro-deoxy-D-glucose positron emission tomography/CT, bilateral bone marrow aspiration and biopsy, lumbar puncture, and ^99^Tc bone scan were also used to examine the involvement of NB in individual metastatic compartments.

### 2.4. Response Criteria

International criteria were used to assess the response to NB treatment [13]. A complete response (CR) was defined as the absence of residual tumor with normal catecholamine levels. A very good partial response (VGPR) was defined as ≥90% reduction in the primary tumor with normal catecholamine levels. A partial response (PR) and a mixed response (MR) were defined based on the comparison of the reduction in the primary and metastatic tumors as ≥50% reduction in both the primary and metastatic lesions and ≥50% reduction only in the primary lesions, respectively. Stable disease (SD) was defined as no new lesion with <25% increase in any existing lesion. Progressive disease (PD) was defined as any new lesion or >25% increase in any measurable lesion.

### 2.5. RNA Isolation

Fresh frozen tissues of 28 patients were used for RNA isolation using the RNeasy Mini Kit (Qiagen, Valencia, CA, USA), according to the manufacturer’s instructions. RNA was quantified using the NanoDrop 8000 UV-Vis Spectrophotometer (NanoDrop Technologies Inc., Wilmington, DE, US) and the Qubit^®^ 3.0 Fluorometer (Life Technologies Inc., Carlsbad, CA, USA). Additionally, RNA quality was assessed using the 4200 TapeStation (Agilent Technologies Inc., Santa Clara, CA, USA). RNA of sufficient quantity and quality was obtained from 25 patients.

### 2.6. RNA Sequencing (RNA-Seq) and Data Analysis

RNA-Seq libraries were prepared according to the TruSeq RNA Sample Prep Kit v2 (Illumina Technologies Inc., San Diego, CA, USA), assayed for quality and quantity, pooled, and then sequenced on the HiSeq 2500 (Illumina Technologies Inc., San Diego, CA, USA) using the 100 bp paired-end mode of the TruSeq Rapid PE Cluster Kit and the TruSeq Rapid SBS Kit v2. The quality control of the FASTQ files which are text files contain the sequencing data and their alignment with the hg19 human reference genome and the reference-guided assembly of transcripts were performed using TopHat version 2.0.6 [16] and Cufflinks version 2.1.1 [17], respectively. The aligned sequence files were re-formatted using SAMtools version 0.1.19 [18]. The gene transcripts in 25 patients were quantified using the count-based method with RSEM software package [19]. A total of 20,180 protein-coding genes were identified, and of these, 15,714 genes that were expressed in at least three patients were used for further analysis. The trimmed mean of M values method was performed for normalization in edgeR [20,21], and voom application from limma [22] was used to transform the normalized counts to log2-counts per million.

### 2.7. Gene Set Variation Analysis (GSVA)

To evaluate the relative activation status of pathways based on metastatic burden, we implemented the GSVA algorithm [23] in our RNA-Seq data. A total of 17,810 annotated gene sets in the Gene Ontology (GO) and Canonical pathways were downloaded from the Molecular Signatures Database (MSigDB v7.0, https://www.gsea-msigdb.org/gsea/msigdb/index.jsp) [24]. Further, we performed GSVA to estimate variation in the pathway activity to identify significantly enriched gene sets of the GO and Canonical pathways. The limma method was performed to identify the differentially expressed pathways.

### 2.8. Statistical Methods

All statistical analyses were performed using the R software version 3.4.2, R (Core Team, Vienna, Austria). The maximally selected log-rank statistics [25] were tested to determine the best possible Curie score cut-point values, which separated patients into good and poor prognostic subgroups in terms of relapse/progression. Clinical characteristics were compared between the two groups using Pearson’s χ^2^ test or Fisher’s exact test for categorical variables and Student’s *t*-test or Wilcoxon rank sum test for continuous variables. The association between Curie score and relapse/progression was evaluated using the non-parametric tests for differences in cumulative incidence, and competing risk regression was evaluated using the Fine and Gray method with death designated as the competing risk. Spearman’s correlation coefficients were calculated for correlation analysis. These tests were analyzed at a significance threshold of *p* < 0.05. The unpaired *t*-test was performed to analyze the differentially expressed genes (DEGs) and GSVA, and a gene/gene set pair was considered differentially expressed at *p* < 0.01. We also calculated the false discovery rate (FDR) to account for multiple testing.

## 3. Results

### 3.1. Characteristics and Outcomes of the Study Population

**Figure 1:**
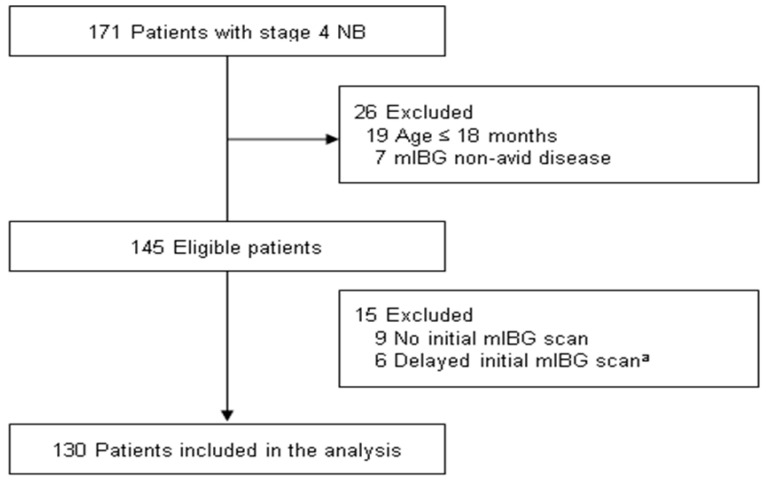
Study flow chart. Abbreviations: NB, neuroblastoma; mIBG, metaiodobenzylguanidine. ^a^ Patients were excluded due to delayed (>30 days after diagnosis) initial mIBG scan.

One hundred and thirty patients with high-risk and metastatic NB (mean (Std) age: 4.5 (2.9) years; 53 (41%) females) were included in the present analysis (Figure 1). The baseline characteristics at diagnosis are summarized in Table 1. Most of the patients (90%) showed an abdominal primary tumor; 22% of the patients had MNA tumors, while 72% had tumors with unfavorable histology according to the International Neuroblastoma Pathology Classification (INPC). At diagnosis, the median Curie score was 12.5 (range, 1–30). The most common site for metastasis was the bone (89%), followed by the bone marrow (71%), and distant lymph nodes (49%). In terms of therapy, 122 (94%) patients received scheduled treatment as per the protocol. Additionally, second HDCT/auto-SCT was suspended for 7 (5%) patients due to treatment-related morbidity, and one patient withdrew from the treatment during induction chemotherapy. Following induction treatment, 51 (39%) patients achieved CR; 29 (22%) patients, VGPR; 41 (32%) patients, PR; two (2%) patients, MR; four (3%) patients, SD; and three (2%) patients, PD. At the time of analysis, 85 patients completed active treatment and underwent assessment for response without experiencing an event or loss to follow-up. Tumor status at the end of the treatment was CR in 45 (53%) patients, VGPR in 17 (20%), PR in 13 (15%), SD in two (2%), and PD in eight (9%). The 5-year event-free survival and the 5-year overall survival for 130 patients were 55.1 ± 5.1% and 69.7 ± 4.7%, respectively.

### 3.2. Prognostic Significance of Metastatic Burden

**Figure 2:**
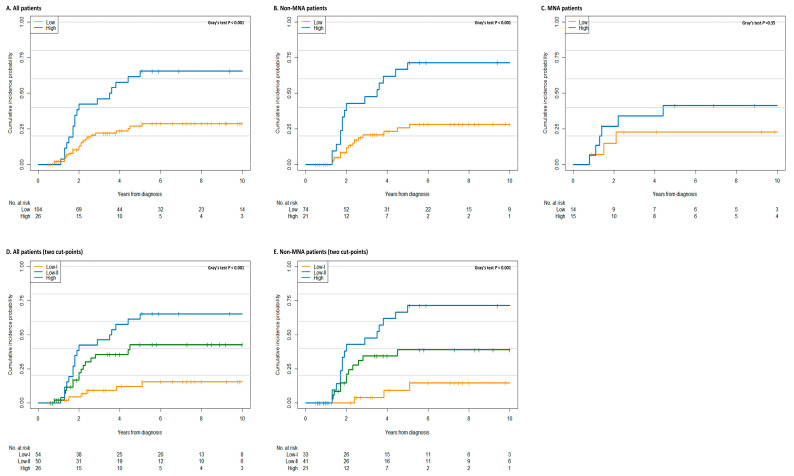
Cumulative incidence rate of relapse/progression based on the metastatic burden, with death as the competing risk. Cumulative incidence curves for relapse/progression based on the best cut-point are illustrated for (**A**) all patients and the (**B**) non-MNA and (**C**) MNA subgroups. The high metastatic burden group shows higher relapse/progression than the low metastatic burden group and the non-MNA subgroup based on a Curie score cut-off value of 26 (*p* < 0.001). However, relapse/progression in patients with MNA tumors is not affected by the degree of metastatic burden at diagnosis even at a Curie score cut-point of 4. Cumulative incidence curves for relapse/progression based on two cut-points are presented for (**D**) all patients and (**E**) the non-MNA group. Death prior to relapse/progression is considered a competing risk. The number of patients at risk is the number of patients alive without relapse/progression. Abbreviations: MNA, *MYCN* amplification.

The importance of metastatic burden as a prognostic factor of high-risk NB was confirmed using the competing risk analysis. The results revealed a progressive and significant association of the Curie scores with relapse/progression (subdistribution hazards ratio (sHR), 1.05; 95% confidence interval (CI), 1.02–1.09; *p* < 0.001). The prognostic significance of the involvement of individual and all metastatic sites was also analyzed (Supplemental Table S2). A Curie score >2, as a cut-point, showed statistical significance for predicting relapse/progression. Based on the maximally selected log-rank statistics, a value of 26 was determined as an optimal cut-point to separate patients into low and high metastatic burden groups (Supplemental Figure S1A). On applying this threshold, the 5-year cumulative incidences of relapse/progression were found to be 27.0 ± 5.2% and 65.4 ± 9.7% for patients with low (*n* = 104) and high (*n* = 26) metastatic burden, respectively, as illustrated in Figure 2A. The Fine and Gray regression model indicated >3-fold higher risk of relapse/progression in patients with high metastatic burden (sHR, 3.06; 95% CI, 1.65–5.65; *p* < 0.001) than in those with low metastatic burden. Using two cut-points to separate patients into three groups with low-I (≤6, *n* = 54), low-II (7–26, *n* = 50), and high (>26, *n* = 26) metastatic burden, we found that the 5-year cumulative incidences of relapse/progression of the three groups were 12.2 ± 5.3%, 42.8 ± 8.5%, and 65.4 ± 9.7%, respectively (Figure 2D). The stratum-specific sHRs increased to 3.43 (95% CI, 1.36–8.63; *p* = 0.009) for the low-II group and 6.32 (95% CI, 2.55–15.63; *p* < 0.001) for the high group, compared with the low-I group.

Further analysis for stratification based on the MNA status was also performed. The significant association of the Curie score, as a continuous variable, with relapse/progression was observed in the non-MNA subgroup (sHR, 1.06; 95% CI, 1.02–1.10; *p* = 0.001), but not in the MNA group (sHR, 1.04; 95% CI, 0.98–1.10; *p* = 0.220). The optimal cut-points and HRs were also found to be influenced by the MNA status. In the non-MNA subgroup, a Curie score of 26 was re-selected (Supplemental Figure S1B). Based on this criterion, patients with high metastatic burden showed significantly higher rates of relapse/progression (sHR, 3.66; 95% CI, 1.83–7.32; *p* < 0.001; Figure 2B). Patients with low metastatic burden were further stratified based on a Curie score of 6. The 5-year cumulative incidences of relapse/progression were 9.2 ± 6.5%, 39.4 ± 9.2%, and 71.4 ± 10.4% for the low-I (≤6, *n* = 33), low-II (7–26, *n* = 41), and high (>26, *n* = 21) metastatic burden subgroups, respectively (*p* < 0.001; Figure 2E). However, the MNA subgroup showed significantly lower optimal cut-point (Curie score of 4; Supplemental Figure S1C) and HRs (sHR, 1.93; 95% CI, 0.51–7.28; *p* = 0.11) than the non-MNA subgroup (Figure 2C).

### 3.3. Distinct Clinical Characteristics Based on the Metastatic Burden

**Figure 3:**
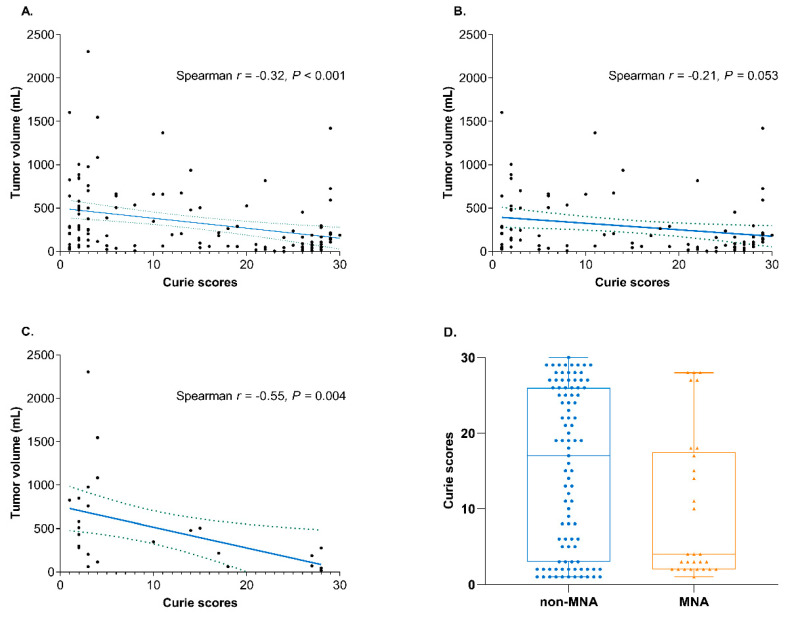
Distinct clinical characteristics associated with the Curie score. Scatterplot indicating the association between the Curie score and primary tumor volume for (**A**) all, (**B**) non-MNA, and (**C**) MNA patients is shown. The solid line represents the regression line, and the concordance correlation coefficient is marked. (**D**) Distribution and comparison of the Curie scores in patients with MNA and non-MNA tumors are given. The line in the middle of each column indicates the median value. Abbreviations: MNA, *MYCN* amplification.

We investigated the differences in clinical features related to the metastatic burden. As a continuous variable, the extent of metastasis was inversely associated with primary tumor volume (Spearman *r* = −0.32, *p* < 0.001), and the MNA tumors showed significantly lower extent of metastasis than the non-MNA tumors (*p* = 0.017; Figure 3). A comparison of clinical features between the high and low metastatic burden groups was performed, as summarized in Table 2. Patients with high metastatic burden showed lesser primary tumor volume than those with low metastatic burden (109 vs. 245 cm^3^; *p* < 0.001). Although MNA tumors showed lower Curie scores, MNA and other cytogenetic statuses were not different between the two groups. Consistent with the observed unfavorable outcomes, patients with high metastatic burden showed higher levels of serum ferritin (400 vs. 224 ng/mL; *p* = 0.025) and 24 h urine VMA (40.0 vs. 14.8 mg/day; *p* = 0.001) than those with low metastatic burden. However, a significant association between early response to chemotherapy and metastatic burden was not observed.

### 3.4. Identification of DEGs and Gene Sets Based on the Metastatic Burden

**Figure 4:**
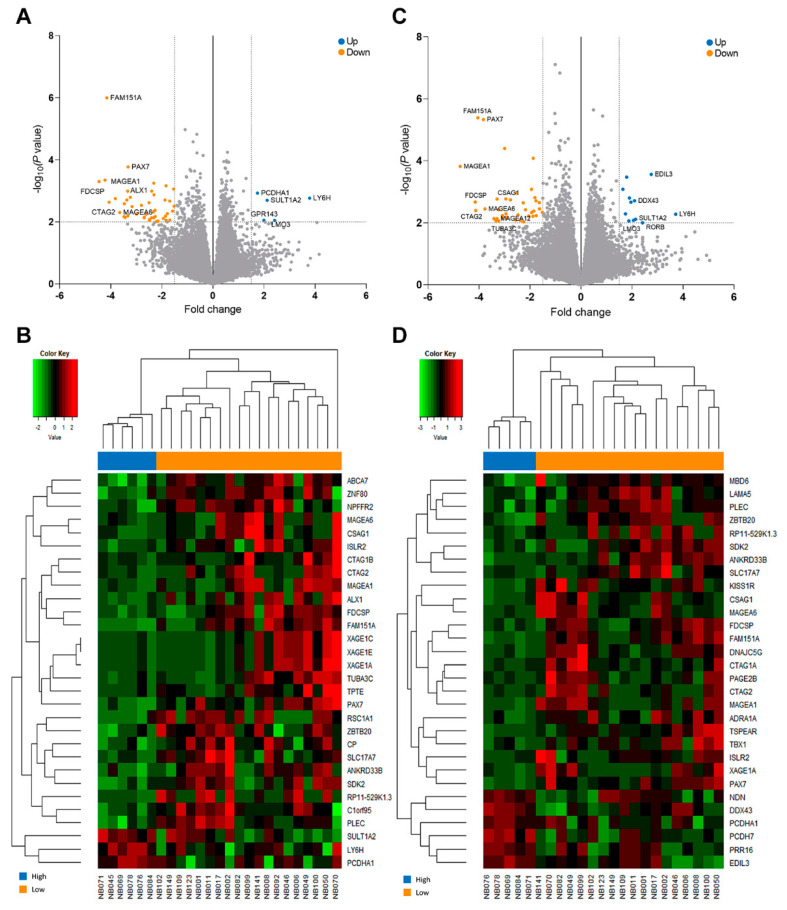
Differential gene expression analysis of patients with high metastatic burden versus that of those with low metastatic burden. Differentially expressed genes between patients with low and high metastatic burden are indicated using volcano plots and heatmaps. Volcano plots show the distribution of the fold change (FC) in gene expression for (**A**) all samples and (**C**) the non-MNA subgroup. Genes with absolute FC ≥ 1.5 and *p* < 0.01 are indicated in blue (higher expression in patients with high metastatic burden than in those with low metastatic burden) and orange (lower expression in patients with high metastatic burden than in those with low metastatic burden). Genes that failed to meet our criteria are represented in gray. Student’s *t*-test was used for the analyses. The heatmap illustrates the top 30 significant genes that were differentially expressed in (**B**) all patients and (**D**) the non-MNA subgroup. Abbreviations: MNA, *MYCN* amplification.

Total RNA-Seq analysis of 25 patients was performed, and the majority of the patients (*n* = 23) presented with non-MNA tumors (Supplemental Table S3). The results indicated 51 DEGs between patients with high and low metastatic burden, based on the selection criteria of log2 fold change (FC) >1.5 and *p* < 0.01 (Supplemental Table S4). Of these, five genes were found to be significantly upregulated and 46 to be downregulated in the high metastatic burden group, compared to the low metastatic burden group, as illustrated using volcano plots in Figure 4A. Figure 4B shows the top 30 significant genes that were differentially expressed in individual patients. We also performed a subgroup analysis based on the MNA status. The analysis of non-MNA tumors indicated 10 upregulated and 40 downregulated DEGs in patients with high metastatic burden, compared to those with low metastatic burden (Figure 4C,D). Statistically pronounced results were achieved in the non-MNA subgroup when compared to all patients (Supplemental Table S5). *EDIL3* and *DDX43* were the most significantly upregulated genes in patients with high metastatic burden, whereas the cancer-testis (CT) antigens, such as *MAGEA1*, were found to show reduced expression in these patients. However, we did not find any significant association between the DEG expression levels and relapse/progression. 

Further, we performed GSVA to identify the relevant biological processes represented by DEGs. The analysis indicated 55 significant gene sets in patients with high metastatic burden, and these distinguished them from patients with low metastatic burden based on the nominal *p* < 0.01, absolute log2 FC > 0.4, and FDR ≤ 0.25 criteria (Supplemental Table S6). The majority of the pathways with strong indication thresholds were found to be related to signaling of various G protein-coupled receptors, protein sorting, ribosomal processes, and amino-acid metabolism. Additional gene sets were differentially expressed according to the metastatic burden in the non-MNA subgroup. A total of 124 gene sets showed statistical significance, and 19 gene sets with FDR < 0.1 were identified (Supplemental Table S7).

## 4. Discussion

This study suggested that patients with extensive NB with distinct clinical and biological features are prone to relapse/progression. The analysis indicated that patients with high metastatic burden showed a substantially elevated risk of relapse/progression when compared to those with low metastatic burden. Moreover, significant differences in clinical characteristics, such as primary tumor volume and biochemical marker levels, were observed between the two groups. Furthermore, using RNA-Seq data, we identified unique gene profiles in the subgroups defined based on the metastatic burden. Fifty-one DEGs and fifty-five gene sets were found to be differentially expressed with modest statistical significance. Additionally, the prognostic effects of metastatic burden were only prominent in patients with non-MNA disease, and genomic differences were affected by the MNA status.

The association of outcomes with the burden of metastatic spread has been examined in previous studies. A study that analyzed the results of the Children’s Oncology Group A3973 protocol, which incorporated single HDCT/auto-SCT as a consolidation treatment, reported that prognosis was not affected by the initial Curie score [5]. The analyses of the International Neuroblastoma Risk Group (INRG) database indicated that the clinical outcomes of patients who underwent HDCT/auto-SCT were not affected by the metastatic burden at diagnosis; however, more than one metastatic system/compartment at diagnosis was associated with prognosis in the pre-HDCT/auto-SCT era [6]. However, conflicting results of the prognostic effects of metastatic burden at diagnosis on patients who received single HDCT/auto-SCT have been reported [8,26]. Furthermore, whether treatment intensification with tandem HDCT/auto-SCT can affect the significance of metastatic burden at diagnosis remains to be evaluated. Most of the patients received tandem HDCT/auto-SCT; therefore, our study provided important clinical implications. Moreover, we observed several cut-points of the Curie score, showing significant statistics for elevated risk of relapse/progression. This suggested that the Curie score at diagnosis could assist in further detailed risk-stratification of high-risk NB patients.

Tumor size has traditionally been considered one of the determinants of prognosis in most malignancies, and hence it has been included in staging systems [27,28]. However, low primary tumor volume was found to be a peculiar characteristic of NB with high metastatic burden in the present study. It is possible that some subtypes of NB can show aggressive behavior despite small primary tumor volume. However, MNA tumors often appeared as large and advanced primary tumors in our prior study [29]. Taken together, these findings suggest that disseminated metastatic NB with small primary tumor volume might be a unique disease entity with enhanced metastatic potential not driven by MNA.

The DEGs with associated genomic pathways identified in this study could partially explain the distinct clinical behaviors. When the analyses were restricted to patients with non-MNA tumors, 50 DEGs were obtained based on the metastatic burden, thereby avoiding the confounding effect of MNA, which is a master transcription factor. Among these genes, *EDIL3, DDX43*, and various CT antigens showed the highest FC. The protein encoded by *EDIL3* is known to be involved in the regulation of cellular migration, and it is over-expressed in several malignancies [30,31]. Moreover, *DDX43*, which encodes the helicase antigen, promotes cellular proliferation, and it is often correlated with poor prognosis [32,33]. In contrast, several groups have reported that aggressive NB downregulates CT antigen expression to preclude recognition by the antigen-specific T-cells [34,35]. These reports provided potential reasoning to our transcriptomic results. However, the corrected *p*-values exceeded 0.05 for most of the DEGs in multiple testing. These values might indicate heterogenous gene expression within each group and analogous gene expression among all groups. Therefore, DEG analysis should be carefully interpreted to avoid undue weightage to these genes as common metastatic driver genes. Additionally, GSVA revealed that most of the proteins encoded by the DEGs were associated with protein sorting, amino acid metabolism, and ribosomal processes. The dysregulation of these processes has been considered a hallmark of cancers with potential to increase cell proliferation and invasion [36,37]. Thus, our findings suggest that inhibition of some of these processes could provide new therapeutic options for NB with disseminated metastasis.

There are several limitations in our study. First, this study was not a controlled prospective study, but instead a retrospective analysis of clinical data. Second, we focused only on patients aged ≥18 months and with mIBG-avid tumors; thus, our patient cohort did not represent the entire high-risk NB patient population. Third, there were a relatively small number of patients with MNA tumors [38], since more patients with MNA tumors were excluded from the analysis when compared to those with non-MNA tumors, based on the eligibility criteria. Fourth, the follow-up duration for some of the patients was relatively short to enable detection of relapse/progression or death, and this might explain the lower relapse/progression rates in this study. Moreover, we did not include GD-2-directed immunotherapy in post-consolidation treatment, and our findings might not be applicable to other study cohorts treated according to the more recent protocols, which include anti-GD-2 immunotherapy. Finally, since the study was not designed to evaluate the comparative RNA-Seq analysis results directly, the RNA samples were readily available in only 20% of the enrolled patients. Due to the small sample size and the high value of the calculated FDR, the genomic differences with metastatic burden could not be generalized to the genomic signature of metastasis in stage 4 high-risk NB. Thus, future studies with a larger sample size should be conducted to determine genomic differences and identify possible therapeutic targets according to the metastatic burden.

In summary, the results of this study indicate that the extent of metastatic burden defined prognostic subgroups of high-risk NB with distinct clinical and genomic features. Metastatic burden at diagnosis exerted prognostic effects in the tandem HDCT/auto-SCT era. Therefore, whether therapeutic escalation is required for patients with high metastatic burden to improve outcomes must be considered. Moreover, the data presented here indicated that the tumors of patients with different extents of metastatic burden were clinically and biologically distinct. Insufficient sample size might limit the identification and analysis of the molecular signature to explain these differences. However, we believe that our findings will contribute to the better understanding of NB behavior and offer insights into additional risk-stratification. Future research with a larger sample size is needed to address the processes governing different extents of metastatic spread.

## Figures and Tables

**Table 1:** Characteristics of the eligible high-risk neuroblastoma patients.

Characteristics	Number (%) ^a^
Sex	
Male	77 (59)
Female	53 (41)
Age at diagnosis, mean ± Std	4.5 ± 2.9
Primary tumor site	
Abdomen/Pelvis	117 (90)
Thorax/Cervix	12 (9)
Unknown	1 (1)
Primary tumor volume ^b^ (cm^3^)	345 (67–510)
*MYCN* status	
Nonamplified	95 (73)
Amplified	29 (22)
Unknown	6 (5)
Cytogenetics	
1p deletion (*n* = 74)	16 (22)
11q deletion (*n* = 79)	23 (29)
17q gain (*n* = 74)	22 (29)
Pathology	
Undifferentiated	15 (12)
Poorly differentiated	65 (50)
Differentiating	22 (17)
Ganglioneuroblastoma	19 (15)
Unknown	9 (7)
INPC	
Favorable	28 (22)
Unfavorable	93 (72)
Unknown	9 (7)
Curie score at diagnosis ^b^	12.5 (3–25)
Metastatic site	
Bone	116 (89)
Bone marrow	92 (71)
Distant lymph node	66 (49)
Lung	14 (11)
CNS	3 (2)
Liver	17 (13)
Skin	5 (4)
Other ^c^	47 (36)

Abbreviations: INPC, International Neuroblastoma Pathology Classification; CNS, central nervous system; Std, standard deviation. ^a^ Percentages were calculated for patients with data available for the given parameter. ^b^ Values are presented as the median (interquartile range). ^c^ Other metastatic sites include all metastatic sites except the bone marrow, bone, liver, lung, CNS, and skin (e.g., pachymeningeal metastases without direction invasion from the cranium, pleural lesions, and muscle).

**Table 2:** Comparison of the characteristics of low and high metastatic burden patients

Variables	Low (*n* = 104)	High (*n* = 26)	*p*-Value
Sex, No. (%)			0.964
Male	61 (59)	16 (62)	
Female	43 (41)	10 (38)	
Age at diagnosis, mean ± Std, years	4.6 ± 3.1	3.9 ± 2.0	0.134
Primary tumor volume (cm^3^) ^b^	245 (1–2305)	109 (9–1421)	0.030
Residual tumor volume (%) ^a, b^	28 (2–98)	31 (10–98)	0.833
Reduction in Curie score, mean ± Std (%) ^c^	44 ± 37	48 ± 36	0.620
Response to induction treatment, No. (%)			
Complete response	42 (40)	9 (35)	
Very good partial response	23 (22)	6 (23)	
Partial response	33 (32)	8 (31)	
Mixed response	1 (1)	1 (4)	
Stable disease	3 (3)	1 (4)	
Progressive disease	2 (2)	1 (4)	
*MYCN* status, No. (%)			0.762
Nonamplified	74 (71)	21 (81)	
Amplified	24 (23)	5 (19)	
Unknown	6 (6)	0	
Cytogenetics, No. (%)			
1p deletion (*n* = 74)	14 (18)	2 (15)	0.920
11q deletion (*n* = 79)	18 (27)	5 (38)	0.633
17q gain (*n* = 79)	18 (27)	4 (33)	0.912
INPC (*n* = 121)			1.000
Favorable	22 (23)	6 (25)	
Unfavorable	75 (77)	18 (75)	
Serum LDH (IU/L) ^b^	1302 (261–10000)	1479 (597–14435)	0.221
Serum ferritin (ng/mL) ^b^	224 (20–3771)	400 (74–1245)	0.025
Serum NSE (ng/mL) ^b^	97 (11–1081)	99 (25–1815)	0.426
24 h urine VMA (mg/day) ^b^	14.8 (0.4–588.4)	40.0 (1.7–100)	0.001

Abbreviations: INPC, International Neuroblastoma Pathology Classification; LDH, lactate dehydrogenase; NSE, neuron-specific enolase; VMA, vanillylmandelic acid; Std, standard deviation. ^a^ Percentage of residual tumor volume was calculated after three cycles of induction chemotherapy, and it reflects early response to chemotherapy. ^b^ Values are presented as the median (range). ^c^ Percentage reduction in Curie score was calculated from diagnosis to after three cycles of induction chemotherapy.

## Supplementary Materials

The following are available online at https://www.mdpi.com/2077-0383/9/9/2730/s1, Figure S1: Optimal cut-point values of the Curie score obtained from maximally selected log-rank statistics. Table S1: Treatment regimens. Table S2: Prognostic significance of individual and different combinations of metastatic sites. Table S3: Characteristics of patients for RNA-Seq analysis. Table S4: Differentially expressed genes between patients with low and high metastatic burden. Table S5: Differentially expressed genes between patients with low and high metastatic burden in the non-*MYCN* amplification (MNA) subgroup. Table S6: Significant gene sets between patients with low and high metastatic burden. Table S7: Significant gene sets between patients with low and high metastatic burden in the non-MNA subgroup.
