# Supplementary material for: Metastatic Burden Defines Clinically and Biologically Distinct Subgroups of Stage 4 High-Risk Neuroblastoma
Source: J Clin Med. 2020 Aug 24;9(9):2730. doi: 10.3390/jcm9092730 (PMC7565784; doi:10.3390/jcm9092730)
Supplement: Supplementary file 1 [file jcm-09-02730-s001.pdf]

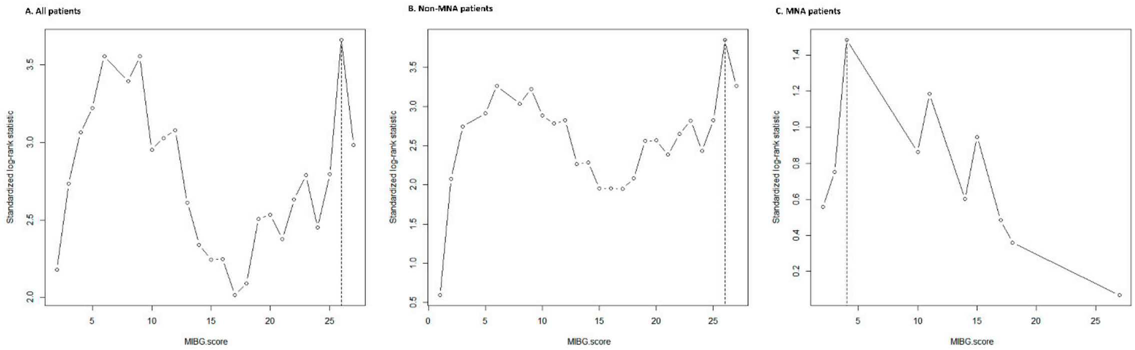

Figure S1. Optimal cut-point values of the Curie score obtained from maximally selected log-rank statistics.

Table S1. Treatment regimens.

Regimen	Drug	Dosage	Schedule	Comments
**Induction**				
CEDC [a]	Cisplatin	60 mg/m^2/day	Day 0	
	Etoposide	100 mg/m^2/day	Day 2, 5	
	Doxorubicin	30 mg/m^2/day	Days 2	
	Cyclophosphamide	30 mg/kg/day	Days 3,4	
ICE[a]	Ifosfamide	1,200 mg/m^2/day	Days 0–4	
	Carboplatin	400 mg/m^2/day	Days 0,1	
	Etoposide	100 mg/m^2/day	Days 0–4	
**Consolidation**				
First HDCT	Carboplatin	60 mg/m^2/day	Days, –7, –6, –5	
	Etoposide	100 mg/m^2/day	Days, –7, –6, –5	
	Cyclophophamide	30 mg/m^2/day	Days, –4, –3, –2	
Second HDCT	Thiotepa	100 mg/m^2/day	Days, –6, –5, –4	
	Melphalan	30 mg/m^2/day	Days, –3, –2	
	TBI	3.33 Gy/day	Days, –3, –2, –1	NB–2004 study
	or ^{131}I-MIBG Tx	12 or 18 mCi/kg	Days –21	NB–2009, 2014 study
**Maintenance**				
Immunotherapy	Interleukin-2		For 5 days every 4 weeks	10 cycles from week 9 of HDCT2
Differentiation Tx	13-cis-retinoic acid	125 mg/m^2/day	For 14 days every 4 weeks	10 cycles from week 9 of HDCT2

MIBG, metaiodobenylguanidine; HDCT, high-dose chemotherapy. [a] The chemotherapy dose was adjusted for patients younger than 24 months of age, i.e., kilogram-based dose for <6 months age, 70% of dose based on the body surface area for 6–11 months of age, 80% dose for 12–17 months of age and dose for 18–23 months of age.

Table S2. Prognostic significance of individual and different combinations of metastatic sites.

Predictor	Unadjusted		Adjusted [a]	
	sHR (95% CI)	*p* value	sHR (95% CI)	*p* value
BM	2.24 (0.94–5.35)	0.071	1.62 (0.62–4.23)	0.320
Bone	1.70 (0.52–5.57)	0.380	1.47 (0.42–5.18)	0.550
Distant lymph nodes	1.40 (0.75–2.60)	0.290	1.28 (0.65–2.49)	0.480
Liver	1.49 (0.72–3.62)	0.380	1.52 (0.73–3.18)	0.260
Skin	0.96 (0.13–7.25)	0.970	1.01 (0.22–4.74)	0.990
Lung [b]	2.32 (1.02–5.28)	0.044	2.22 (0.92–5.35)	0.076
CNS [c]	1.68 (0.62–4.61)	0.310	1.32 (0.25–6.87)	0.740
Other	2.85 (1.51–5.39)	0.001	2.34 (1.12–4.90)	0.023
MSI [d]	1.56 (1.22–2.01)	<0.001	2.30 (1.17–4.54)	0.012

Abbreviations: sHR, subdistributional hazard ratio; CI, confidence interval; BM, bone marrow; CNS, central nervous system, MSI, metastatic site index. [a] Adjusted for *MYCN* amplification and modified Curie score. [b] Direct invasion from thoracic tumors or pleural involvement was excluded. [c] Only parenchymal or leptomeningeal lesions are considered CNS metastases. [d] Metastatic site index (MSI) is a score based on the number of metastatic system/compartments involved (potentially up to 8: bone marrow, bone, distant lymph nodes, liver, skin, lung, CNS and other).

Table S3. Characteristics of patients for RNA-Seq analysis.

Characteristics	RNA sample (*n* = 25)	Others (*n* = 105)	*p*-value
Sex. No. (%)			
Male	20 (80)	57 (54)	0.034
Age at diagnosis, mean ± SD	4.9 ± 2.6	4.4 ± 3.0	0.458
Primary tumor site, No (%)			0.860
Abdomen/Pelvis	23 (92)	94 (90)	
Primary tumor volume (cm^3), mean ± SD	325 ± 396	350 ± 393	0.777
MYCN status			
Nonamplified	23 (92)	72 (73)	0.077
Amplified	2 (8)	27 (20)	
Cytogenetics, No. (%)			
1p deletion	19 (79)	44 (80)	1.000
11q deletion	11 (46)	12 (22)	0.059
17q gain	6 (26)	16 (29)	1.000
INPC			1.000
Favorable	6 (25)	22 (23)	
Unfavorable	18 (75)	75 (77)	
Curie score at diagnosis, mean ± SD	14.3 ± 11.0	13.5 ± 10.7	0.733
Response to induction treatment, No. (%)			0.166
Complete response	6 (24)	45 (43)	
Very good partial response	5 (20)	24 (23)	
Partial response	13 (52)	28 (27)	
Mixed response	0	2 (2)	
Stable disease	0	4 (4)	
Progressive disease	1 (4)	2 (2)	

Abbreviations: INPC, International Neuroblastoma Pathology Classification; SD, standard deviation.

Table S4. Differentially expressed genes between patients with low and high metastatic burden.

	Gene Name	Log_2(fold change)	*P* value	FDR
**Down-regulated**	*FAM151A*	-4.153784656	1.01E-06	0.015892
	PAX7	-3.323674818	0.00017	0.209025
	FDCSP	-4.459497745	0.000504	0.255283
	MAGEA1	-4.225267699	0.000452	0.255283
	SDK2	-2.318223576	0.000568	0.264813
	SLC17A7	-1.838057332	0.000689	0.292807
	CSAG1	-3.338727913	0.001031	0.305655
	RP11-529K1.3	-2.396452916	0.001023	0.305655
	PLEC	-1.543008079	0.000881	0.305655
	CTAG2	-4.063645168	0.002333	0.32981
	ALX1	-3.8211702	0.001792	0.32981
	XAGE1C	-3.371049802	0.001979	0.32981
	XAGE1E	-3.369198581	0.001978	0.32981
	XAGE1A	-3.236800008	0.001618	0.32981
	CP	-2.502895198	0.002406	0.32981
	ISLR2	-2.310967438	0.00134	0.32981
	ZBTB20	-1.880308778	0.001976	0.32981
	C1orf95	-1.693227193	0.002143	0.32981
	TUBA3C	-3.469798412	0.00262	0.333735
	RSC1A1	-3.167977549	0.003216	0.343777
	NPFFR2	-2.801977346	0.002939	0.343777
	ABCA7	-1.521702671	0.003126	0.343777
	TPTE	-2.269247269	0.00423	0.37347
	ANKRD33B	-1.575120709	0.004514	0.380294
	ZNF80	-2.324200658	0.004721	0.390455
	MAGEA6	-3.651662116	0.004974	0.399244
	CTAG1B	-1.78515239	0.005408	0.409445
	HLA-DRB5	-2.994055715	0.005635	0.411874
	CDH1	-2.613623571	0.00567	0.412125
	MAGEA12	-3.331860724	0.006389	0.432767
	STRA6	-1.857751499	0.006518	0.435164
	GP1BA	-3.483631324	0.006755	0.436819
	KEL	-2.25973468	0.006747	0.436819
	IGFL4	-2.688758243	0.007036	0.438892
	MAGEA3	-3.434153605	0.007189	0.441152
	IL17REL	-2.313892101	0.007324	0.442071
	ENTHD1	-2.689621917	0.007386	0.442725
	EHF	-2.404255566	0.00741	0.442725
	SCN5A	-1.942418932	0.007741	0.447231
	CTAG1A	-2.48748255	0.008384	0.462104
	KIF21B	-1.836564468	0.00882	0.469643
	PLCH2	-1.819999509	0.008973	0.469643
	ADRA1A	-1.689082933	0.008757	0.469643
	MYBPC2	-2.484164428	0.009026	0.469658
	OR7D2	-2.147383601	0.009514	0.483196
	KCNC3	-2.209992386	0.009666	0.483344
**Up-regulated**	*PCDHA1*	1.740932287	0.001185894	0.329810196
	SULT1A2	2.119078124	0.002010513	0.329810196
	LY6H	3.784993658	0.001724316	0.329810196
	GPR143	1.996851721	0.008914896	0.469642767
	LMO3	2.4134251	0.008936518	0.469642767

Table S5. Differentially expressed genes between patients with low and high metastatic burden in the non-*MYCN* amplification (MNA) subgroup.

	Gene Name	Log_2 (fold change)	*P* value	FDR
**Down-regulated**	*FAM151A*	-4.04175	4.09E-06	0.01051
	PAX7	-3.82958	4.68E-06	0.01051
	TSPEAR	-2.99112	4.00E-05	0.037003
	ANKRD33B	-1.8745	8.28E-05	0.065027
	MAGEA1	-4.74031	0.000152	0.077148
	KISS1R	-1.94125	0.00084	0.178306
	RP11-529K1.3	-2.53041	0.001111	0.206196
	ZBTB20	-1.80925	0.001542	0.230113
	CSAG1	-3.29065	0.00171	0.234916
	CTAG1A	-2.94693	0.001698	0.234916
	DNAJC5G	-2.76385	0.001816	0.234916
	SLC17A7	-1.76354	0.001965	0.239075
	FDCSP	-4.14753	0.002137	0.246939
	SDK2	-2.2666	0.002268	0.250713
	MBD6	-1.63333	0.002232	0.250713
	MAGEA6	-3.76836	0.003591	0.281457
	PAGE2B	-3.22185	0.003752	0.281457
	TBX1	-2.1758	0.003797	0.281457
	PLEC	-1.62616	0.003574	0.281457
	CTAG2	-4.12586	0.004034	0.292099
	ISLR2	-1.84972	0.004197	0.29355
	ADRA1A	-1.93233	0.004633	0.306003
	LAMA5	-1.51653	0.004625	0.306003
	XAGE1A	-2.93906	0.005124	0.317753
	TGIF2LX	-2.46893	0.005573	0.318284
	XAGE1C	-3.08077	0.00587	0.320152
	XAGE1E	-3.07882	0.00587	0.320152
	C1orf95	-1.75586	0.005869	0.320152
	PLCH2	-2.04674	0.006041	0.322401
	CTAG1B	-1.88515	0.006206	0.322942
	SYCE1	-3.06809	0.006802	0.328035
	SPDYE2	-2.92582	0.00687	0.328035
	TUBA3C	-3.41744	0.007324	0.336524
	MAGEA12	-3.29575	0.007351	0.336786
	CP	-2.56143	0.008165	0.349597
	SLC9A3	-2.37399	0.008436	0.354459
	ALX1	-3.33167	0.008684	0.355366
	RSC1A1	-3.23033	0.008696	0.355366
	TPTE	-2.25218	0.009376	0.363788
**Up-regulated**	*PRR16*	1.642109	0.000829	0.178306
	RCAN2	1.744963	0.005168	0.317753
	NDN	1.790537	0.000338	0.120579
	C10orf107	1.875407	0.008721	0.355366
	PCDHA1	1.900439	0.001583	0.231292
	PCDH7	1.955574	0.002171	0.247406
	LMO3	2.057861	0.008351	0.352314
	DDX43	2.101648	0.001946	0.238933
	SULT1A2	2.141738	0.007732	0.343123
	EDIL3	2.754512	0.000274	0.107751
	LY6H	3.71531	0.005291	0.317753

Table S6. Significant gene sets between patients with low and high metastatic burden.

	Gene Set Name	log2FC	*P* value	FDR
Down-regulated	GO_ARRESTIN_FAMILY_PROTEIN_BINDING	-0.549	0.0000000	0.0004
	GO_POSITIVE_REGULATION_OF_ACTION_POTENTIAL	-0.495	0.0000115	0.0345
	GO_ADP_RIBOSYLATION_FACTOR_BINDING	-0.608	0.0000424	0.0548
	GO_SPINAL_CORD_ASSOCIATION_NEURON_DIFFERENTIATION	-0.477	0.0000461	0.0548
	GO_AP_2_ADAPTOR_COMPLEX_BINDING	-0.502	0.0001952	0.1010
	GO_POSITIVE_REGULATION_OF_ACTIN_FILAMENT_BASED_MOVEMENT	-0.548	0.0003439	0.1278
	BIOCARTA_ION_PATHWAY	-0.563	0.0004024	0.1306
	GO_NEURAL_PLATE_DEVELOPMENT	-0.411	0.0004782	0.1364
	GO_REGULATION_OF_AUTOPHAGOSOME_MATURATION	-0.428	0.0005492	0.1433
	GO_NEGATIVE_REGULATION_OF_LOW_DENSITY_LIPOPROTEIN_PARTICLE_CLEARANCE	-0.571	0.0007795	0.1795
	GO_ESTABLISHMENT_OF_LYMPHOCYTE_POLARITY	-0.472	0.0007698	0.1795
	BIOCARTA_PPARG_PATHWAY	-0.425	0.0007505	0.1795
	GO_CYTOSKELETAL_ANCHORING_AT_NUCLEAR_MEMBRANE	-0.578	0.0012505	0.1880
	GO_MEMBRANE_DEPOLARIZATION_DURING_AV_NODE_CELL_ACTION_POTENTIAL	-0.476	0.0010827	0.1880
	GO_REGULATION_OF_NEURON_PROJECTION_ARBORIZATION	-0.458	0.0010631	0.1880
	GO_RESPONSE_TO_IMMUNE_RESPONSE_OF_OTHER_ORGANISM_INVOLVED_IN_SYMBIOTIC_INTERACTION	-0.466	0.0017664	0.2079
Up-regulated	GO_RIBOSOME_DISASSEMBLY	0.635	0.0000048	0.0289
	GO_CYSTEINE_METABOLIC_PROCESS	0.421	0.0000103	0.0345
	GO_RIBONUCLEOPROTEIN_COMPLEX_DISASSEMBLY	0.428	0.0000180	0.0431
	GO_SIGNAL_RECOGNITION_PARTICLE_ENDOPLASMIC_RETICULUM_TARGETING	0.647	0.0000220	0.0441
	GO_NUCLEOTIDE_EXCISION_REPAIR_DNA_DAMAGE_RECOGNITION	0.455	0.0000325	0.0548
	GO_CYCLOSPORIN_A_BINDING	0.477	0.0000548	0.0548
	GO_TRNA_SPLICING_LIGASE_COMPLEX	0.534	0.0000519	0.0548
	GO_PROTEIN_REFOLDING	0.408	0.0000669	0.0574
	GO_MALATE_METABOLIC_PROCESS	0.546	0.0001577	0.0902
	GO_PROLINE_BIOSYNTHETIC_PROCESS	0.547	0.0001503	0.0902
	GO_ANDROGEN_BIOSYNTHETIC_PROCESS	0.490	0.0002371	0.1095
	GO_SIGNAL_RECOGNITION_PARTICLE	0.479	0.0003834	0.1278
	REACTOME_ATF6_ATF6_ALPHA_ACTIVATES_CHAPERONES	0.414	0.0005047	0.1364
	GO_FATTY_ACID_ALPHA_OXIDATION	0.526	0.0005113	0.1364
	GO_EGO_COMPLEX	0.590	0.0004892	0.1364
	GO_MALATE_DEHYDROGENASE_ACTIVITY	0.506	0.0008154	0.1795
	GO_PREFOLDIN_COMPLEX	0.515	0.0008256	0.1795
	GO_PURINE_RIBONUCLEOSIDE_SALVAGE	0.564	0.0008822	0.1795

GO_PROTEASOME_REGULATORY_PARTICLE_BASE_SUBCOMPLEX	0.522	0.0009681	0.1845
GO_PROTEIN_DENEDDYLATION	0.448	0.0009937	0.1864
GO_MITOCHONDRIAL_RNA_MODIFICATION	0.424	0.0013180	0.1880
GO_HYPEROSMOTIC_SALINITY_RESPONSE	0.450	0.0013330	0.1880
GO_TRANSLATION_TERMINATION_FACTOR_ACTIVITY	0.464	0.0012943	0.1880
GO_AMP_BIOSYNTHETIC_PROCESS	0.472	0.0013778	0.1880
GO_RNA_IMPORT_INTO_MITOCHONDRION	0.532	0.0011609	0.1880
GO_ESCRT_COMPLEX_DISASSEMBLY	0.419	0.0013945	0.1881
REACTOME_SUMO_IS_CONJUGATED_TO_E1_UBA2:SAE1	0.470	0.0015154	0.1936
GO_POSITIVE_REGULATION_OF_PROTEIN_MONOUBIQUITINATION	0.488	0.0015229	0.1936
REACTOME_ALPHA_OXIDATION_OF_PHYTANATE	0.512	0.0016586	0.1991
GO_REGULATION_OF_CILIUM_BEAT_FREQUENCY	0.404	0.0018452	0.2116
BIOCARTA_AHSP_PATHWAY	0.521	0.0018507	0.2116
GO_GONADAL_MESODERM_DEVELOPMENT	0.563	0.0020124	0.2246
GO_7S_RNA_BINDING	0.531	0.0021160	0.2277
HALLMARK_OXIDATIVE_PHOSPHORYLATION	0.412	0.0022386	0.2326
GO_SUCKLING_BEHAVIOR	0.463	0.0022517	0.2326
GO_FREE_UBIQUITIN_CHAIN_POLYMERIZATION	0.443	0.0023919	0.2361
GO_MITOCHONDRIAL_TRNA_METHYLATION	0.459	0.0024582	0.2361
KEGG_PROTEIN_EXPORT	0.496	0.0024565	0.2361
GO_THIOREDOXIN_PEROXIDASE_ACTIVITY	0.533	0.0024212	0.2361

Table S7. Significant gene sets between patients with low and high metastatic burden in the non-MNA subgroup.

	Gene Set Name	log2FC	*P* value	FDR
Down-regulated	GO_ADP_RIBOSYLATION_FACTOR_BINDING	-0.665	0.0000051	0.013806
	GO_ARRESTIN_FAMILY_PROTEIN_BINDING	-0.539	0.0000057	0.013806
	GO_NEURAL_PLATE_DEVELOPMENT	-0.509	0.0000058	0.013806
	GO_ATP_BINDING_CASSETTE_ABC_TRANSPORTER_COMPLEX	-0.750	0.0000126	0.016390
	BIOCARTA_ION_PATHWAY	-0.648	0.0000137	0.016390
	GO_POSITIVE_REGULATION_OF_ACTION_POTENTIAL	-0.517	0.0000111	0.016390
	GO_CONVERGENT_EXTENSION_INVOLVED_IN_ORGANOGENESIS	-0.412	0.0000209	0.018852
	GO_SPINAL_CORD_ASSOCIATION_NEURON_DIFFERENTIATION	-0.495	0.0001254	0.053772
	BIOCARTA_PITX2_PATHWAY	-0.418	0.0001229	0.053772
	GO_POSITIVE_REGULATION_OF_ACTIN_FILAMENT_BASED_MOVEMENT	-0.588	0.0001698	0.057253
	GO_ROUNDABOUT_BINDING	-0.493	0.0001593	0.057253
	GO_RRNA_GUANINE_METHYLTRANSFERASE_ACTIVITY	-0.582	0.0002157	0.057759
	GO_REGULATION_OF_AUTOPHAGOSOME_MATURATION	-0.461	0.0002266	0.057759
	GO_MODULATION_OF_MICROTUBULE_CYTOSKELETON_INVOLVED_IN_CEREBRAL_CORTEX_RADIAL_GLIA_GUIDED_MIGRATION	-0.541	0.0004903	0.084072
	GO_AP_2_ADAPTOR_COMPLEX_BINDING	-0.514	0.0005029	0.085026
	GO_NEURAL_PLATE_MORPHOGENESIS	-0.497	0.0005295	0.085895

GO_CYTOSKELETAL_ANCHORING_AT_NUCLEAR_MEMBRANE	-0.638	0.0006059	0.092976
REACTOME_NEGATIVE_REGULATION_OF_TCF_DEPENDENT_SIGNALING_BY_DVL_INTERACTING_PROTEINS	-0.724	0.0006749	0.097676
BIOCARTA_PPARG_PATHWAY	-0.435	0.0006967	0.099567
GO_GATOR1_COMPLEX	-0.506	0.0008073	0.108970
GO_NEGATIVE_REGULATION_OF_LOW_DENSITY_LIPOPROTEIN_PARTICLE_CLEARANCE	-0.544	0.0010435	0.129140
GO_NEGATIVE_REGULATION_OF_HISTONE_DEACETYLATION	-0.670	0.0011672	0.137368
GO_NEURONAL_ACTION_POTENTIAL_PROPAGATION	-0.433	0.0011883	0.138265
GO_ANTEROGRADE_DENDRITIC_TRANSPORT	-0.548	0.0013104	0.138910
BIOCARTA_PELP1_PATHWAY	-0.455	0.0012601	0.138910
GO_RADIAL_GLIA_GUIDED_MIGRATION_OF_PURKINJE_CELL	-0.631	0.0016921	0.153882
GO_KICSTOR_COMPLEX	-0.561	0.0018619	0.161962
GO_U3_SNORNA_BINDING	-0.493	0.0021965	0.179613
GO_CHONDROBLAST_DIFFERENTIATION	-0.455	0.0022483	0.179613
GO_NUCLEAR_MIGRATION_ALONG_MICROTUBULE	-0.608	0.0023689	0.181758
GO_NEURONAL_SIGNAL_TRANSDUCTION	-0.595	0.0024868	0.182274
GO_NEGATIVE_REGULATION_OF_SINGLE_STRANDED_VIRAL_RNA_REPLICATION_VIA_DOUBLE_STRANDED_DNA_INTERMEDIATE	-0.437	0.0024524	0.182274
GO_NEGATIVE_REGULATION_OF_EXECUTION_PHASE_OF_APOPTOSIS	-0.430	0.0025981	0.183570
GO_SOFT_PALATE_DEVELOPMENT	-0.523	0.0029839	0.194961
GO_REGULATION_OF_NEURON_PROJECTION_ARBORIZATION	-0.4	0.002	0.19

		47	9291	4961
	GO_ESTABLISHMENT_OF_LYMPHOCYTE_POLARITY	-0.4<br>37	0.003<br>1652	0.19<br>7505
	REACTOME_STAT5_ACTIVATION	-0.4<br>25	0.003<br>1656	0.19<br>7505
	GO_L_LYSINE_TRANSMEMBRANE_TRANSPORTER_ACTIVITY	-0.5<br>29	0.004<br>6780	0.22<br>7671
	GO_GASTRIC_EMPTYING	-0.4<br>62	0.004<br>6171	0.22<br>7671
	GO_PHOSPHATIDYLINOSITOL_TRANSPORTER_ACTIVITY	-0.4<br>30	0.005<br>1467	0.23<br>4910
	GO_POSITIVE_REGULATION_OF_CREB_TRANSCRIPTION_FACTOR_ACTIVITY	-0.4<br>51	0.005<br>3580	0.24<br>0182
	GO_MEMBRANE_DEPOLARIZATION_DURING_AV_NODE_CELL_ACTION_POTENTIAL	-0.4<br>40	0.005<br>5009	0.24<br>2769
Up-regu<br>lated	GO_CYSTEINE_METABOLIC_PROCESS	0.47<br>5	0.000<br>0007	0.00<br>7829
	GO_TRNA_SPLICING_LIGASE_COMPLEX	0.60<br>2	0.000<br>0031	0.01<br>3806
	GO_ENDOSOME_TRANSPORT_VIA_MULTIVESICULAR_BODY_SORTING_PATHWAY	0.40<br>2	0.000<br>0111	0.01<br>6390
	GO_REGULATION_OF_GLUCOCORTICOID_RECEPTOR_SIGNALING_PATHWAY	0.62<br>0	0.000<br>0087	0.01<br>6390
	REACTOME_ATF6_ATF6_ALPHA_ACTIVATES_CHAPERONE_GENES	0.47<br>8	0.000<br>0152	0.01<br>6627
	GO_CYCLOSPORIN_A_BINDING	0.52<br>7	0.000<br>0178	0.01<br>7796
	GO_RIBOSOME_DISASSEMBLY	0.66<br>8	0.000<br>0354	0.02<br>8313
	GO_RIBONUCLEOPROTEIN_COMPLEX_DISASSEMBLY	0.46<br>9	0.000<br>0424	0.02<br>9919
	GO_LATE_ENDOSOME_TO_VACUOLE_TRANSPORT	0.45<br>7	0.000<br>0573	0.03<br>6179
	GO_SIGNAL_RECOGNITION_PARTICLE_ENDOPLASMIC_RETICULUM_TARGETING	0.66<br>2	0.000<br>0603	0.03<br>6208
	GO_NUCLEOTIDE_EXCISION_REPAIR_DNA_DAMAGE_RECOGNITION	0.46<br>1	0.000<br>0728	0.03<br>9702

KEGG_PROTEIN_EXPORT	0.593	0.0000703	0.039702
GO_PROTEIN_PEPTIDYL_PROLYL_ISOMERIZATION	0.422	0.0001675	0.057253
REACTOME_ATF6_ATF6_ALPHA_ACTIVATES_CHAPERONES	0.463	0.0001598	0.057253
GO_NEGATIVE_REGULATION_OF_MEMBRANE_POTENTIAL	0.512	0.0001717	0.057253
GO_ANDROGEN_BIOSYNTHETIC_PROCESS	0.514	0.0001467	0.057253
GO_PROLINE_BIOSYNTHETIC_PROCESS	0.546	0.0001641	0.057253
GO_COPI_VESICLE_COAT	0.442	0.0002372	0.057759
GO_POSTTRANSLATIONAL_PROTEIN_TARGETING_TO_ENDOPLASMIC_RETICULUM_MEMBRANE	0.445	0.0002295	0.057759
GO_AMP_BIOSYNTHETIC_PROCESS	0.566	0.0002406	0.057759
GO_3_HYDROXYACYL_COA_DEHYDROGENASE_ACTIVITY	0.575	0.0002185	0.057759
GO_RNA_IMPORT_INTO_MITOCHONDRION	0.595	0.0001872	0.057759
GO_PROTEIN_UFMYLATION	0.596	0.0002382	0.057759
REACTOME_WAX_AND_PLASMALOGEN_BIOSYNTHESIS	0.579	0.0002572	0.059363
GO_SIGNAL_RECOGNITION_PARTICLE	0.510	0.0003730	0.072317
REACTOME_SYNTHESIS_SECRETION_AND_INACTIVATION_OF_GLUCOSE_DEPENDENT_INSULINOTROPIC_POLYPEPTIDE_GIP	0.514	0.0003735	0.072317
GO_LATE_ENDOSOME_TO_VACUOLE_TRANSPORT_VIA_MULTIVESICULAR_BODY_SORTING_PATHWAY	0.552	0.0003735	0.072317
GO_COPII_VESICLE_COAT	0.518	0.0003962	0.075490
REACTOME_CYTOCHROME_C_MEDIATED_APOPTOTIC_RESPONSE	0.417	0.0004159	0.076815
GO_PROTEIN_PRENYLTRANSFERASE_ACTIVITY	0.41	0.000	0.08

	4	4708	2331
GO_REGULATION_OF_ENDOPLASMIC_RETICULUM_TUBULAR_NETWORK_ORGANIZATION	0.590	0.0004723	0.082331
GO_ENDOPLASMIC_RETICULUM_TUBULAR_NETWORK_MEMBRANE	0.549	0.0005295	0.085895
GO_POSITIVE_REGULATION_OF_PROTEIN_MONOUBIQUITINATION	0.557	0.0006038	0.092976
GO_PREFOLDIN_COMPLEX	0.540	0.0006754	0.097676
GO_PROTEIN_POLYUFMYLATION	0.738	0.0007412	0.104675
GO_MITOCHONDRIAL_ACETYL_COA_BIOSYNTHETIC_PROCESS_FROM_PYRUVATE	0.717	0.0008352	0.108970
GO_MALATE_METABOLIC_PROCESS	0.463	0.0010435	0.129140
GO_OXIDOREDUCTASE_ACTIVITY_ACTING_ON_PAIRED_DONORS_WITH_INCORPORATION_OR_REDUCTION_OF_MOLECULAR_OXYGEN_REDUCED_ASCORBATE_AS_ONE_DONOR_AND_INCORPORATION_OF_ONE_ATOM_OF_OXYGEN	0.416	0.0012310	0.138910
REACTOME_ZINC_EFFLUX_AND_COMPARTMENTALIZATION_BY_THE_SLC30_FAMILY	0.472	0.0012980	0.138910
GO_PURINE_RIBONUCLEOSIDE_SALVAGE	0.596	0.0013240	0.138910
GO_FATTY_ACID_ALPHA_OXIDATION	0.572	0.0013501	0.139715
GO_CELLULAR_LIPID_BIOSYNTHETIC_PROCESS	0.428	0.0015798	0.147745
GO_PROTEASOME_REGULATORY_PARTICLE_BASE_SUBCOMPLEX	0.539	0.0015368	0.147745
GO_EGO_COMPLEX	0.638	0.0016542	0.152748
GO_MEDIUM_CHAIN_FATTY_ACID_CATABOLIC_PROCESS	0.514	0.0018394	0.161168
GO_POSITIVE_REGULATION_OF_MAINTENANCE_OF_SISTER_CHROMATID_COHESION	0.420	0.0022100	0.179613
GO_GLYCEROL_ETHER_BIOSYNTHETIC_PROCESS	0.437	0.0022232	0.179613
GO_REGULATION_OF_PROTEIN_MONOUBIQUITINATION	0.440	0.0022973	0.179613

GO_HYPEROSMOTIC_SALINITY_RESPONSE	0.46<br>3	0.002<br>2832	0.17<br>9613
GO_PROTEIN_SERINE_THREONINE_PHOSPHATASE_INHIBITOR_ACTIVITY	0.50<br>4	0.002<br>2915	0.17<br>9613
REACTOME_GLUCURONIDATION	0.53<br>3	0.002<br>1609	0.17<br>9613
GO_OMEGA_HYDROXYLASE_P450_PATHWAY	0.45<br>9	0.002<br>4075	0.18<br>1758
REACTOME_SYNTHESIS_OF_16_20_HYDROXYEICOSATETRAENOIC_ACIDS_HETE	0.45<br>9	0.002<br>4075	0.18<br>1758
GO_PROTEIN_NEDDYLATION	0.60<br>1	0.002<br>4767	0.18<br>2274
GO_PROTEIN_GERANYLGERANYLTRANSFERASE_ACTIVITY	0.46<br>3	0.002<br>6016	0.18<br>3570
GO_THIOREDOXIN_PEROXIDASE_ACTIVITY	0.56<br>2	0.002<br>6559	0.18<br>4952
GO_PROTEIN_DENEDDYLATION	0.41<br>4	0.002<br>7894	0.19<br>1340
REACTOME_GOLGI_CISTERNAE_PERICENTRIOLAR_STACK_REORGANIZATION	0.41<br>7	0.002<br>7799	0.19<br>1340
GO_REGULATION_OF_CENTRIOLE_ELONGATION	0.52<br>0	0.002<br>8599	0.19<br>3843
GO_KINOCILIUM	0.42<br>8	0.003<br>0386	0.19<br>5750
REACTOME_SUMO_IS_CONJUGATED_TO_E1_UBA2:SAE1	0.45<br>6	0.003<br>1095	0.19<br>7505
GO_HOST_CELL_CYTOPLASM	0.46<br>2	0.003<br>1756	0.19<br>7505
GO_FREE_UBIQUITIN_CHAIN_POLYMERIZATION	0.48<br>6	0.003<br>2248	0.19<br>7505
GO_RIBOSOMAL_LARGE_SUBUNIT_BINDING	0.43<br>4	0.003<br>4777	0.20<br>3639
GO_NEDD8_TRANSFERASE_ACTIVITY	0.54<br>7	0.003<br>6609	0.20<br>8275
GO_SEQUENCE_SPECIFIC_MRNA_BINDING	0.44<br>4	0.003<br>7370	0.20<br>8647
GO_ENDOSOME_TO_LYSOSOME_TRANSPORT_VIA_MULTIVESICULAR_BODY_SORTING_PATHWAY	0.51	0.003	0.20

	8	7268	8647
REACTOME_TRAFFICKING_OF_MYRISTOYLATED_PROTEINS_TO_THE_CILIUM	0.459	0.0037941	0.209880
GO_REGULATION_OF_CILIUM_BEAT_FREQUENCY	0.460	0.0038867	0.212942
REACTOME_ALPHA_OXIDATION_OF_PHYTANATE	0.546	0.0040242	0.216623
GO_TRANSLATION_TERMINATION_FACTOR_ACTIVITY	0.434	0.0041667	0.217834
GO_MITOCHONDRIAL_PROTON_TRANSPORTING_ATP_SYNTHASE_COMPLEX_ASSEMBLY	0.655	0.0042828	0.222555
GO_PURINE_NUCLEOSIDE_CATABOLIC_PROCESS	0.457	0.0044642	0.225176
REACTOME_VIF_MEDIATED_DEGRADATION_OF_APOBEC3G	0.415	0.0046592	0.227671
GO_GLYCOSIDE_METABOLIC_PROCESS	0.431	0.0047536	0.227671
HALLMARK_OXIDATIVE_PHOSPHORYLATION	0.442	0.0046257	0.227671
GO_REGULATION_OF_CILIUM_BEAT_FREQUENCY_INVOLVED_IN_CILIARY_MOTILITY	0.597	0.0047553	0.227671
GO_MITOCHONDRIAL_TRNA_METHYLATION	0.456	0.0048197	0.227777
BIOCARTA_AHSP_PATHWAY	0.562	0.0050309	0.231022
GO_POSTTRANSLATIONAL_PROTEIN_TARGETING_TO_MEMBRANE_TRANSLOCATION	0.696	0.0050027	0.231022
GO_7S_RNA_BINDING	0.518	0.0053623	0.240182
REACTOME_PROPIONYL_COA_CATABOLISM	0.670	0.0054207	0.241895
